# Improved on-treatment fibrosis-4 during antiviral therapy and lower hepatocellular carcinoma risk in cirrhotic patients with hepatitis B

**DOI:** 10.1038/s41598-023-36668-2

**Published:** 2023-06-09

**Authors:** Joo Hyun Oh, Yewan Park, Myung Ji Goh, Dong Hyun Sinn, Sang Bong Ahn, Wonseok Kang, Geum-Youn Gwak, Yong-Han Paik, Moon Seok Choi, Joon Hyeok Lee, Seung Woon Paik

**Affiliations:** 1 Department of Medicine, Samsung Medical Center, Sungkyunkwan University School of Medicine, 81 Irwon-ro, Gangnam-gu, Seoul, 06351 Korea; 2 Department of Medicine, Nowon Eulji Medical Center, Eulji University School of Medicine, Seoul, 01830 Korea; 3 Department of Medicine, Kyung Hee University School of Medicine, Seoul, 02447 Korea

**Keywords:** Hepatitis, Liver

## Abstract

Normalization of serum alanine aminotransferase (ALT) levels is one of the goals of hepatitis B treatment. However, ALT levels in cirrhosis patients might be normal or mildly elevated regardless of ongoing inflammation. Therefore, we examined whether on-treatment ALT and other potential on-treatment indicators could be clinical surrogates of antiviral therapy in HBV-related cirrhosis. A total of 911 patients with HBV-related liver cirrhosis who started treatment with entecavir or tenofovir were analyzed. At 1 year of antiviral therapy, we evaluated ‘ALT normalization’, ‘undetectable serum HBV DNA’, ‘fibrosis-4 (FIB-4) index improvement’, and ‘serum HBeAg loss’ as potential biomarkers for HCC development. During 6.6 (3.8–10.2) years of follow-up, 222 patients (24.3%) newly developed HCC. Undetectable HBV DNA levels at 1 year were observed in 667 patients (73.2%), and the HCC incidence was significantly lower in this population (adjusted hazard ratio (HR) 0.66, 95% CI 0.50–0.87). Improvement of the FIB-4 index (< 3.25) was associated with a lower risk of HCC in 478 patients with an elevated FIB-4 index (adjusted HR 0.59, 95% CI 0.55–0.82). However, there was no significant difference in HCC risk between those with and without normalization of ALT levels (*p* = 0.39) among those with elevated ALT levels or between those with and without HBeAg seroconversion (*p* = 0.55) among HBeAg-positive patients. Therefore, on-treatment FIB-4 levels at 1 year are clinically useful surrogates of antiviral therapy for HBV-related cirrhosis patients.

## Introduction

Chronic hepatitis B virus (CHB) is a leading cause of liver cirrhosis, hepatocellular carcinoma (HCC), and liver-related mortality globally^1^. By lowering hepatic inflammation and fibrosis, the treatment goals of CHB infection are to reduce the risk of cirrhosis and HCC and enhance survival^2^. Although these goals can be fulfilled by the early and total eradication of the hepatitis B virus (HBV) from hepatocytes, complete eradication of HBV is likely to be unachievable with current antiviral therapies (AVT). Nucloes(t)ide analogues (NAs) such as entecavir and tenofovir are not virucidal but virustatic. Although HBV replication might be effectively suppressed, covalently closed circular DNA could persist in hepatocytes and continue to induce low-grade inflammation^3^. Patients taking NAs are still at risk of cirrhosis and HCC. Therefore, regular monitoring after starting NAs is required.

HBV deoxyribonucleic acid (DNA) levels are commonly used to assess AVT response. Virological response (VR) is defined as ‘a reduction in serum HBV DNA to undetectable HBV DNA on a real-time polymerase chain reaction assay’^2^. Unfortunately, the use of HBV DNA is inconvenient since it requires at least 1–3 days after blood sampling for HBV DNA, requiring patients to visit the hospital twice. Moreover, HBV DNA levels cannot be used as on-treatment surrogates after achieving VR. Other biomarkers, including serum alanine aminotransferase (ALT), hepatitis B envelope antigen (HBeAg), and hepatitis B surface antigen (HBsAg), are also associated with virological response, the development of HCC, and death^4,5^. Recently, the significance of ALT normalization has been emphasized. Patients with CHB have a decreased risk of hepatic events if their ALT levels are normal over the first 12 months of NAs^6,7^. However, ALT levels can be normal or mildly elevated in cirrhosis patients with necroinflammation^8^. A decrease in ALT level may not suggest remission of inflammation in these populations^9^. Besides, several other factors such as alcohol, drugs, and non-alcoholic fatty liver disease (NAFLD), can influence ALT levels^10^. For these reasons, current guidelines recommend AVT for cirrhotic patients with active HBV replication, regardless of ALT levels^2^.

The hypothesis was that on-treatment markers, which capture the dynamic changes during therapy, may offer additional prognostic information beyond baseline assessments. However, the ability of normalization of ALT to predict HCC development in patients with CHB cirrhosis remains unclear. The on-treatment FIB-4 score, a simple and easily accessible marker that can be calculated during routine follow-up visits, has shown promise in predicting the risk of HCC in cirrhotic patients receiving antiviral therapy. Therefore, the aim of this study was to explore the potential of on-treatment markers, specifically the on-treatment FIB-4 score, as a tool to assess the risk of HCC development in cirrhotic patients receiving antiviral therapy.

## Results

### Baseline characteristics and HCC development during follow-up

Baseline characteristics of 911 patients are shown in Table 1. Their median age was 51.7 years. There were 581 (63.8%) males. Most (79.7%) patients were treated with entecavir. During a median follow-up of 6.6 years (interquartile range 3.8–10.1 years), 222 (24.3%) patients were newly diagnosed with HCC. The cumulative incidence rate of HCC was 8.7% at 3 years and 17.8% at 5 years. Results of the comparison of baseline characteristics between those with HCC and those without HCC are shown in Supplementary Table S1.

**Table 1:** Characteristics of study population at baseline and 1-year after antiviral therapy (n = 911).

	Baseline	At 1 year
Age (year)	51.7 (46.5–57.4)	–
Male	580 (63.8)	–
Body mass index (kg/m^2^)	24.3 (22.6–26.3)	–
Hypertension	90 (9.9)	–
Diabetes mellitus	113 (12.4)	–
Dyslipidemia	71 (7.8)	–
Platelet (× 10^3^/UL)	117 (87–150)	–
Albumin (g/dL)	4.0 (3.6–4.3)	–
AST (U/dL)	53 (41–83)	–
PT INR	1.12 (1.05–1.24)	–
Nucleot(s)ide analogue		
Entecavir	726 (79.7)	
Tenofovir	185 (20.3)	
HBeAg positivity	367 (56.5)	327 (50.4)
HBeAg loss/seroconversion	–	55/367
HBV DNA (log_10_ IU/ml)	5.6 (4.8–6.5)	1.5 (1.3–2.0)
Undetectable levels (< 12 IU/L)		667 (73.2)
ALT (IU/L)	54 (38–89)	
Normal ALT levels	164 (18.0)	482 (52.9)
Elevated ALT levels	747 (82.0)	429 (47.1)
Normalization of ALT level	–	370/747 (49.5)
FIB-4	3.41 (2.17–5.93)	
≤ 3.25	433 (47.5)	560 (61.5)
> 3.25	478 (52.5)	351 (38.5)
Improvement of FIB-4 ≤ 3.25	–	163/478 (34.1)

Values were expressed as median (quartile) or number (%).

### On-treatment biomarkers and the risk of HCC

**Figure 1:**
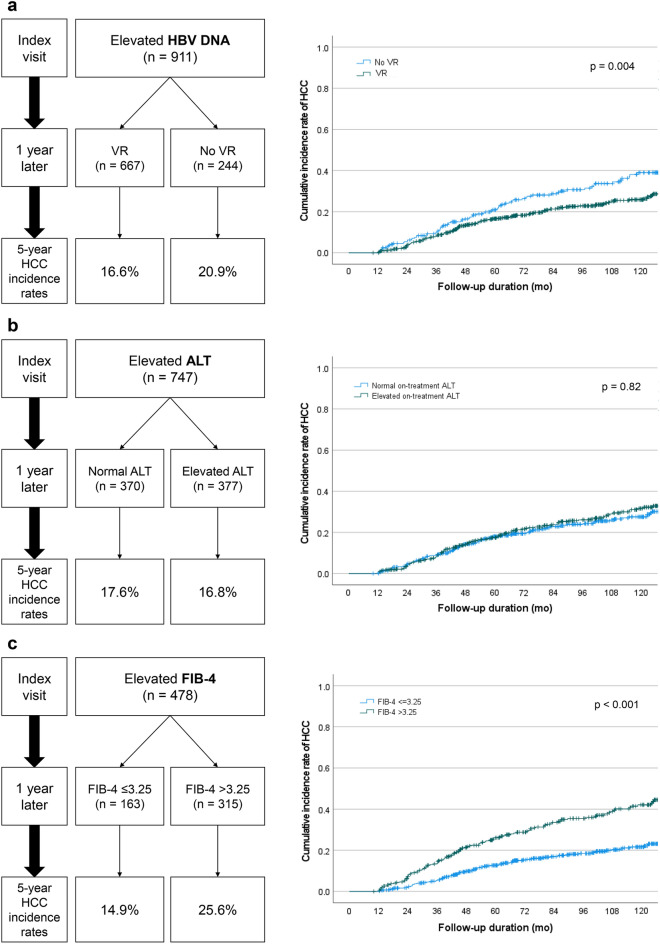
(**a**) Response to antiviral therapy according to on-treatment HBV DNA. (**b**) Response to antiviral therapy according to on-treatment ALT. (**c**) Response to antiviral therapy according to on-treatment Fibrosis-4.

At baseline, all patients showed elevated HBV DNA levels (> 2000 IU/m, median 5.6 log_10_IU/mL), and 667 of 911 patients (73.2%) achieved VR at 1 year of entecavir (ETV)/tenofovir (TDF) therapy. The 5-year incidence rate of HCC was lower in patients who achieved VR compared to those who did not (16.6% vs. 20.9%, *p* = 0.004) (Fig. 1a). In multivariate analysis, VR was an independent factor associated with the development of HCC (adjusted HR 0.66, 95% CI 0.50–0.87) (Table 2, Supplementary Table S2). Subgroup analysis was performed for patients who achieved VR, and in this subgroup, the improvement of FIB-4 at 1 year remained an independent prognostic factor for HCC development in both univariate and multivariate analyses (unadjusted HR 0.47, 95% CI 0.34–0.66 and adjusted HR 0.49, 95% CI 0.35–0.70) (Supplementary Table S3).

**Table 2:** Association between on-treatment biomarkers and future HCC risk.

Endpoint	Number at risk	Proportion achieved endpoint at 1 year	Un-adjusted HR (95% CI)	*p* value	Adjusted HR (95% CI)	*p* value
Undetectable HBV DNA levels	911	667 (73.2)	0.66 (0.50–0.87)	0.004	0.66 (0.50–0.87)	0.004
HBeAg loss/seroconversion	367	55 (14.9)	1.16 (0.70–1.93)	0.55	1.17 (0.70–1.94)	0.53
Normalization of ALT	747	370 (49.5)	0.88 (0.65–1.17)	0.39	0.82 (0.61–1.10)	0.19
Improvement of FIB-4 (≤ 3.25)	478	163 (34.1)	0.59 (0.40–0.86)	0.007	0.55 (0.38–0.82)	0.003

At baseline, HBeAg was positive in 367 (56.5%) patients. After 1 year of ETV/TDF therapy, HBeAg loss/seroconversion was achieved in 14.9% (55/367) of HBeAg positive patients. There was no significant difference in the 5-year incidence rates of HCC between patients who achieved HBeAg loss/seroconversion and those who did not among HBeAg positive patients (19.7% vs. 20.1%, *p* = 0.55) (Table 2, Supplementary Table S4).

Since HBsAg testing is infrequently conducted in clinical practice, it was not feasible to include it as a routine marker in this study. Upon reviewing the 1 year HBsAg follow-up data, only 87 out of 911 patients had HBsAg follow-up data available, and among them, five patients showed HBsAg loss. The five patients did not develop HCC during the follow-up period, while the remaining 82 patients developed HCC in 15 patients. A subgroup analysis was conducted, excluding the participants who experienced HBsAg loss. In the multivariate analysis, the results of undetectable HBV DNA levels and improvement of FIB-4 at 1 year remained consistent (Supplementary Table S5).

At baseline, 747 (82%) patients showed elevated ALT. One year after ETV/TDF therapy, 370 out of 747 patients (49.5%) achieved normalization of ALT levels. The 5-year cumulative incidence rate of HCC was 17.6% in patients with normalized on-treatment ALT and 16.8% in patients with elevated on-treatment ALT (*p* = 0.39) (Fig. 1b). ALT normalization was not found to be an independent factor associated with HCC development (adjusted HR 0.82, 95% CI 0.61–1.10) (Table 2, Supplementary Table S6) among patients with elevated ALT levels.

At baseline, 478 (52.5%) patients had an elevated FIB-4 index (> 3.25). Improvement of the FIB-4 index (≤ 3.25) was observed in 34.1% (163/478) of patients after 1 year of ETV/TDF therapy. The 5-year cumulative incidence rate of HCC was lower in patients with an improved FIB-4 index at 1 year compared to those who did not achieve improvement (14.9% vs. 25.6%, *p* = 0.006) (Fig. 1c). Improvement in the FIB-4 index at 1 year was an independently protective factor associated with decreased risk of HCC development (adjusted HR 0.55, 95% CI 0.38–0.82) (Table 2, Supplementary Table S7).

The modified PAGE-B score was used to stratify 911 patients into low risk (≤ 8), intermediate (9–12), and high (≥ 13) risk groups^11^. While the high-risk group had a significantly higher 5-year cumulative incidence rate of HCC (24.7%), there was no significant difference in HCC incidence rates between the low and intermediate risk groups (6.2% and 11.3%, respectively, *p* = 0.71). We also analyzed the prognostic value of on-treatment FIB-4 in each risk group and found that improvement in FIB-4 levels was associated with a significant decrease in HCC development in both intermediate and high-risk patients after 1 year of antiviral therapy (Supplementary Table S8).

## Discussion

Entecavir and tenofovir are first-line recommended antiviral therapies for people with chronic HBV infection^2^. These therapies can inhibit HBV replication, normalize serum ALT levels, induce HBeAg loss, and even improve liver fibrosis^12^. In the present cohort of HBV-related liver cirrhosis patients with elevated HBV DNA at baseline, 73.2% achieved VR. Among patients with elevated ALT levels at baseline, 370 (49.5%) patients achieved normalization of ALT levels at 1 year of NAs therapy. In addition, 34.1% of patients with high FIB-4 showed significant improvement in their FIB-4 index in 1 year following AVT. VR (HBV DNA < 12 IU/mL) and improvement of FIB-4 index (≤ 3.25) at 1 year of AVT were significantly associated with a decreased risk of HCC (adjusted HR 0.66, 95% CI 0.50–0.87; and adjusted HR 0.55, 95% CI 0.38–0.80, respectively), indicating that VR and improvement of FIB-4 index could be used as on-treatment surrogates associated with incident HCC. On the other hand, ALT normalization and HBeAg loss at 1 year of AVT were not associated with a reduced risk of HCC.

Active HBV replication is known to damage the liver parenchyma and induce the development of liver cirrhosis and HCC^13^. Although AVT can suppress HBV replication and improve histological necroinflammation and fibrosis, not all patients respond to AVT. The response rates of AVT, especially entecavir and tenofovir, are approximately 68–83% after 48 weeks of NAs therapy^3^. Our data are consistent with previous studies showing that the risk of HCC is higher for those who do not achieve VR compared to those who achieving VR^14,15^. This phenomenon was more evident in patients with cirrhosis^16^. Insufficient suppression of viral replication might lead to HBV DNA integration, genomic instability due to mutation, and necroinflammation^17^.

Normalization of ALT levels is regarded as a treatment goal of AVT^2^. Several studies have shown that normalization of ALT levels after AVT is linked to a decreased risk of HCC in CHB patients^7,18^. However, in this study, there was no significant difference in HCC risk between patients with normalized ALT levels and those without. This might be related to the characteristics of the cohort. In the present study, we only assessed patients with HBV-related liver cirrhosis. In these patients, who have necroinflammation, ALT levels can be normal or mildly elevated^1^. In addition, a variety of factors can affect serum ALT levels, such as NAFLD, and drug induced liver injury^19^. This suggests that ALT normalization at 1 year is insufficient to reflect the risk of HCC in liver cirrhosis patients.

AVT not only effectively inhibits HBV replication but also improves liver fibrosis^12,20^. By managing inflammation, suppressing excessive extracellular matrix synthesis, and regulating cellular viability, patients under treatment with NAs can experience changes in fibrosis^21^. In this study, we used the serum biomarker FIB-4^22,23^ to estimate the change in liver fibrosis. The FIB-4 index is widely accepted as a non-invasive tool to estimate liver fibrosis. With a FIB-4 index threshold of 3.25, its specificity for predicting significant fibrosis was 95.2% in CHB patients^22^. Kim et al. have demonstrated that a relatively short period of AVT, about 1–2 years, can lower liver stiffness measurements in CHB patients^24^. The regression of fibrosis induced by 1 year of NAs might lower the risk of HCC. Therefore, FIB-4 measurement at 1 year can offer an easy assessment of liver fibrosis and help identify cirrhosis patients at risk of HCC.

Accurate stratification of underlying risk is necessary for effective surveillance of HCC in patients with CHB cirrhosis. This study shows that patients with persistently high FIB-4 values after receiving AVT for 1 year are at significantly higher risk for HCC than those with improved FIB-4 levels. This finding could aid in tailoring HCC surveillance for patients with CHB cirrhosis. Similar findings have been observed in chronic hepatitis C (CHC) and NAFLD patients. Studies have consistently shown that longitudinal changes in FIB-4 may serve as a useful tool for predicting the risk of HCC. For example, a retrospective study of 1,325 CHB patients treated with entecavir found that those with a significant decrease in FIB-4 score at 1 year of therapy had the lowest risk of developing HCC during follow-up^25^. Similarly, a study of 343 patients with CHC found that patients with a decrease in FIB-4 index after direct-acting agent therapy had a significantly lower risk of developing liver-related events, including HCC, compared to those with a smaller decrease or increase in FIB-4 index^26^. Finally, a study of 455 NAFLD patients found that those with an increase in FIB-4 of at least 0.3 had a significantly higher risk of cirrhosis and HCC than those with stable or decreasing FIB-4 levels^27^. This study has several limitations. Firstly, this was a retrospective cohort study of Korean patients with CHB cirrhosis who were infected with genotype C. Thus, its applicability to other ethnic groups is uncertain. Secondly, further procedures to detect fibrosis, including transient elastography, magnetic resonance elastography, and a liver biopsy, were not performed. Additional analysis using these assessments is required to validate our observations. Thirdly, there were other unmeasured risk factors for HCC, including a familial history of HCC, alcohol consumption, and other metabolic risk factors. Despite these limitations, the strength of this data comes from a large cohort study with a long-term follow-up period.

In conclusion, we have demonstrated that patients with CHB who achieve VR or improvement of FIB-4 within the first year of AVT have a lower risk of HCC. Therefore, HBV-related cirrhosis patients should be closely monitored if they have consistently elevated HBV DNA or the FIB-4 index, even if they are receiving high genetic barrier NAs therapy.

## Patients and methods

### Study design, setting and participants

**Figure 2:**
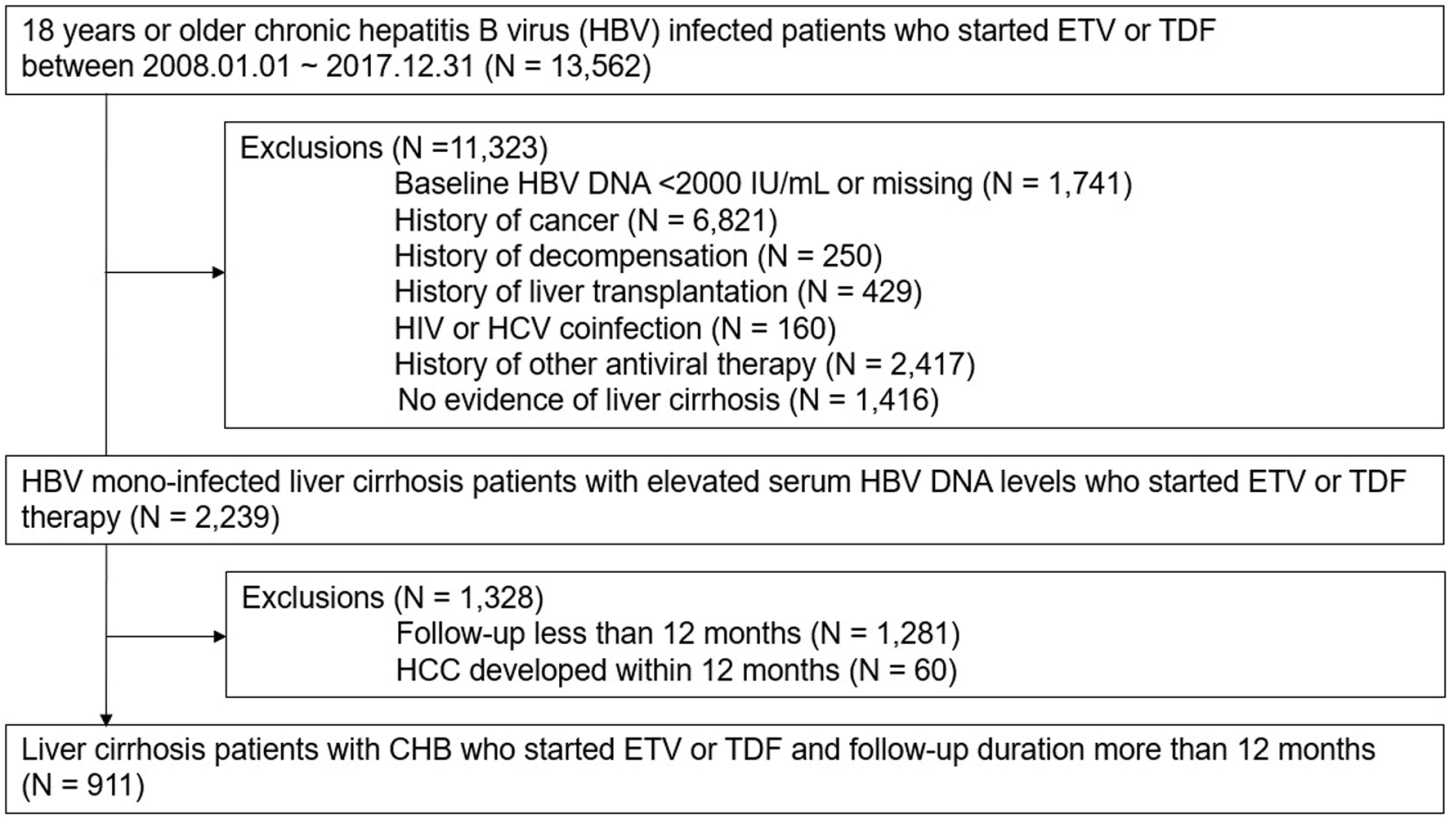
Participants.

This is a multi-center, retrospective cohort study conducted at Samsung Medical Center and Nowon Eulji Medical Center, Seoul, Korea. Between January 1, 2008, and December 31, 2017, a total of 13,562 adult patients infected with chronic hepatitis B who initiated AVT using ETV or TDF were identified. Patients who met any of the following exclusion criteria were excluded from the analysis: (1) baseline HBV DNA < 2000 IU/mL or missing; (2) history of any cancer; (3) history of hepatic decompensation; (4) history of liver transplantation; (5) human immunodeficiency virus or hepatitis C virus coinfection; (6) history of AVT (interferon, lamivudine, adefovir, telbivudine, and clevudine); (7) no evidence of liver cirrhosis. Liver cirrhosis was clinically defined as the presence of nodular liver surface or caudate lobe hypertrophy on liver imaging studies (ultrasonography, CT, or MRI), thrombocytopenia (< 150,000/µL), splenomegaly, or varices^28^. After excluding patients with a follow-up of less than 12 months or HCC development within 1 year of treatment, a total of 911 liver cirrhosis patients with CHB who started ETV or TDF and had a follow-up duration of more than 12 months were analyzed (Fig. 2). The study was conducted in accordance with the principles of the Declaration of Helsinki. The Institutional Review Board (IRB) of Samsung Medical Center (IRB 2021-02-103) and Nowon Eulji Medical Center (IRB 2021-08-005) reviewed and approved the protocol. As the study used only de-identified data routinely collected during hospital visits, the requirement to obtain informed patient consent was waived by the IRB.

### Definitions of outcome and variables

The primary endpoint was the development of HCC during follow-up. Follow-up duration was measured from the date of starting AVT to the date of HCC diagnosis or the last follow-up, whichever came first (reference date: August 7th, 2021). The diagnosis of HCC was based on regional HCC guidelines during the study period^29,30^.

The following baseline and on-treatment variables were collected from the electronic medical records of each center. Collected baseline variables were age, gender, body mass index, co-morbidities (hypertension, diabetes mellitus, dyslipidemia), serum platelet, albumin, aspartate aminotransferase (AST), ALT, prothrombin time, HBeAg, HBsAg, HBV DNA levels, and results of liver imaging studies. On-treatment variables were: HBV DNA, HBeAg, HBsAg, AST, ALT, and platelet count at 1 year of AVT. Normal ALT levels were defined as ≤ 34 IU/L for men and ≤ 30 IU/L for women^2^. The FIB-4 index was calculated using the following formula: age (years) * AST (IU/mL)/[platelet count (10^9^/L) * ALT (IU/mL)^1/2^]. Based on the published cut-off for the FIB-4 index, elevated FIB-4 was defined as a FIB-4 index > 3.25^31,32^.

We defined four on-treatment responses at 1 year of AVT: (1) VR, defined as undetectable serum HBV DNA level (< 12 IU/mL) at 1 year; (2) HBeAg loss, defined as HBeAg loss and/or seroconversion at 1 year for HBeAg positive patients; (3) normalization of ALT levels, defined as normal ALT levels at 1 year in patients with elevated ALT levels (> 34 IU/L for men and > 30 IU/L for women) at baseline; and (4) improvement of FIB-4, defined as FIB-4 index ≤ 3.25 at 1 year in patients with elevated FIB-4 index (> 3.25) at baseline.

### Subgroup analysis

We conducted a subgroup analysis on patients who achieved VR since most patients are expected to achieve VR after antiviral treatment due to the use of high genetic barrier drugs. In addition, HBsAg loss has been suggested as a significant predictor of HCC development. To account for this, we performed a separate subgroup analysis that excluded participants who experienced HBsAg loss.

### Statistical analysis

Baseline and on-treatment values are expressed as median (interquartile range) or number (%). Characteristics of patients with and without developing HCC were compared using the Student’s t-test or Chi-square test, as appropriate. Cumulative incidence rate curves were plotted using the Kaplan–Meier method. The log-rank test was used to compare curves between patients with and without on-treatment response to AVT at 1 year (VR, HBeAg loss for those with HBeAg positive patients, ALT normalization for those with elevated ALT levels, and improvement of FIB-4 for those with elevated FIB-4). A multivariate adjusted cox-regression analysis was performed to determine whether on-treatment response at 1 year was an independent factor associated with HCC development. For adjustment, variables with *p* < 0.05 in univariate cox-regression analysis were used.

Statistical significance was defined as *p* < 0.05. All statistical analyses were performed using SPSS program version 28.0.

### Ethical approval

The Institutional Review Board at Samsung Medical Center and Nowon Eulji Medical Center reviewed and approved the study protocol.

## Supplementary Information

Supplementary Tables.

## Data Availability

The data that support the findings of this study are available on request from the corresponding author. The data is not publicly available due to privacy or ethical restrictions.
