# Supplementary material for: Improved on-treatment fibrosis-4 during antiviral therapy and lower hepatocellular carcinoma risk in cirrhotic patients with hepatitis B
Source: Sci Rep. 2023 Jun 9;13:9443. doi: 10.1038/s41598-023-36668-2 (PMC10256734; doi:10.1038/s41598-023-36668-2)
Supplement: Supplementary file 1 — Supplementary Tables. [file 41598_2023_36668_MOESM1_ESM.docx]

**Supplementary Table S1.** Characteristics of patients with and without developing HCC (n = 911).

|  | **Without developing HCC (n = 689)** | **With developing HCC**  **(n = 222)** | **p value** |
| --- | --- | --- | --- |
| **Age** (year) | 51.2 (45.8-56.5)) | 53.4 (48.2-59.5) | <0.001 |
| **Male** | 418 (60.7) | 163 (73.4) | <0.001 |
| **Body mass index** (kg/m2) | 24.3 (22.4-26.4) | 24.3 (22.7-26.0) | 0.71 |
| **Hypertension** | 64 (9.3) | 26 (11.7) | 0.29 |
| **Diabetes mellitus** | 83 (12.0) | 30 (13.5) | 0.56 |
| **Dyslipidemia** | 55 (8.0) | 16 (7.2) | 0.70 |
| **Platelet** (x10^3^/UL) | 120 (92-154) | 107 (72-181) | <0.001 |
| **Albumin** (g/dL) | 4.0 (3.7-4.4) | 3.9 (3.4-4.2) | <0.001 |
| **AST** (U/dL) | 52 (40-83) | 57 (43-82) | 0.17 |
| **PT INR** | 1.11 (1.05-1.21) | 1.17 (1.08-1.30) | 0.025 |
| **Nucleot(s)ide analogue** |  |  | <0.001 |
| **Entecavir** | 529 (76.8) | 197 (88.7) |  |
| **Tenofovir** | 160 (23.2) | 25 (11.3) |  |
| **HBeAg positivity** | 261 (37.8) | 106 (47.7) | 0.05 |
| **HBeAg loss/seroconversion** | 37 (14.2) | 18 (17.0) | 0.49 |
| **HBV DNA (log_10_ IU/ml)** | 5.6 (4.7-6.5) | 5.8 (4.9-6.5) | 0.18 |
| **Undetectable levels (<12 IU/L)**  **at one year** | 522 (75.8) | 145 (65.3) | 0.002 |
| **ALT** (IU/L) | 55 (38-95) | 53 (36-80) | 0.026 |
| **Normal ALT levels** | 125 (18.1) | 39 (17.6) | 0.84 |
| **Elevated ALT levels** | 564 (81.9) | 183 (82.4) | 0.002 |
| **Normalization of ALT level**  **at one year** | 284/564 (50.4) | 86/183 (47.0) | 0.43 |
| **FIB-4** | 3.18 (2.03-5.30) | 4.45 (2.81-7.29) | <0.001 |
| **≤3.25** | 353 (51.2) | 80 (36.0) | <0.001 |
| **>3.25** | 336 (48.8) | 142 (64.0) |  |
| **Improvement of FIB-4 ≤3.25**  **at one year** | 127/336 (37.8) | 36/142 (25.3) | 0.009 |

Values were expressed as median (quartile) or number (%). Abbreviations:

**Supplementary Table S2.** Factor associated with hepatocellular carcinoma among patients with detectable HBV DNA levels (n = 911).

|  | **Un-adjusted HR (95% CI)** | **p value** | **Adjusted HR**  **(95% CI)** | **p value** |
| --- | --- | --- | --- | --- |
| **Age** | 1.03 (1.02-1.05) | <0.001 | 1.04 (1.02-1.06) | <0.001 |
| **Sex, female** | 0.61 (0.45-0.83) | 0.001 | 0.46 (0.34-0.64) | <0.001 |
| **Body mass index** | 0.98 (0.93-1.04) | 0.63 |  |  |
| **Hypertension** | 1.10 (0.73-1.66) | 0.62 |  |  |
| **Diabetes** | 1.18 (0.80-1.74) | 0.38 |  |  |
| **Dyslipidemia** | 1.02 (0.61-1.71) | 0.91 |  |  |
| **TDF (vs. ETV)** | 0.63 (0.41-0.96) | 0.034 | 0.70 (0.46-1.07) | 0.10 |
| **HBeAg positivity** | 1.27 (0.97-1.65) | 0.07 |  |  |
| **Elevated ALT** | 0.92 (0.65-1.30) | 0.65 |  |  |
| **FIB-4** | 1.06 (1.03-1.09) | <0.001 | 1.06 (1.03-1.09) | <0.001 |
| **Undetectable HBV DNA levels at one year** | 0.66 (0.50-0.87) | 0.004 | 0.66 (0.50-0.87) | 0.004 |

**Supplementary Table S3.** Factor associated with hepatocellular carcinoma among patients who achieved VR (n=667).

|  | **Un-adjusted HR (95% CI)** | **p value** | **Adjusted HR**  **(95% CI)** | **p value** |
| --- | --- | --- | --- | --- |
| **Age** | 1.04 (1.02-1.06) | <0.001 | 1.04 (1.02-1.06) | <0.001 |
| **Sex, female** | 0.57 (0.39-0.82) | 0.003 | 0.40 (0.28-0.59) | <0.001 |
| **Body mass index** | 0.98 (0.92-1.04) | 0.60 |  |  |
| **Hypertension** | 1.37 (0.96-1.97) | 0.08 |  |  |
| **Diabetes** | 1.35 (0.86-2.10) | 0.18 |  |  |
| **Dyslipidemia** | 1.40 (0.77-2.53) | 0.26 |  |  |
| **TDF (vs. ETV)** | 0.69 (0.42-1.13) | 0.14 |  |  |
| **HBeAg positivity** | 1.25 (0.90-1.74) | 0.18 |  |  |
| **HBV DNA (log_10_ IU/ml)** | 1.08 (0.93-1.24) | 0.28 |  |  |
| **Elevated ALT level** | 0.89 (0.58-1.36) | 0.59 |  |  |
| **Improvement of FIB-4 index (<3.25) at one year** | 0.47 (0.34-0.66) | <0.001 | 0.49 (0.35-0.70) | <0.001 |

**Supplementary Table S4.** Factor associated with hepatocellular carcinoma among patients with HBeAg positivity (n = 367).

|  | **Un-adjusted HR (95% CI)** | **p value** | **Adjusted HR**  **(95% CI)** | **p value** |
| --- | --- | --- | --- | --- |
| **Age** | 1.04 (1.02-1.07) | <0.001 | 1.04 (1.02-1.07) | <0.001 |
| **Sex, female** | 0.63 (0.41-0.95) | 0.029 |  |  |
| **Body mass index** | 0.98 (0.90-1.06) | 0.61 |  |  |
| **Hypertension** | 1.10 (0.59-2.05) | 0.76 |  |  |
| **Diabetes** | 1.24 (0.71-2.14) | 0.43 |  |  |
| **Dyslipidemia** | 0.96 (0.50-1.85) | 0.91 |  |  |
| **TDF (vs. ETV)** | 0.78 (0.41-1.48) | 0.46 |  |  |
| **Elevated ALT** | 0.94 (0.77-1.15) | 0.60 |  |  |
| **HBV DNA (log_10_ IU/ml)** | 1.01 (0.87-1.18) | 0.81 |  |  |
| **FIB-4 index** | 1.02 (0.98-1.07) | 0.22 |  |  |
| **HBeAg loss/seroconversion at one year** | 1.16 (0.70-1.93) | 0.55 | 1.17 (0.70-1.94) | 0.53 |

**Supplementary Table S5.** Subgroup analysis excluding HBsAg loss patients: Factors associated with hepatocellular carcinoma among patients with detectable HBV DNA levels (n = 906), among patients with HBeAg positivity (n = 364), and patients with elevated serum alanine aminotransferase levels (n = 745)

|  | **Detectable HBV DNA levels** | | **HBeAg positivity** | | **Elevated serum ALT** | | **Elevated FIB-4** | |
| --- | --- | --- | --- | --- | --- | --- | --- | --- |
|  | **Adjusted HR**  **(95% CI)** | **p value** | **Adjusted HR**  **(95% CI)** | **p value** | **Adjusted HR**  **(95% CI)** | **p value** | **Adjusted HR**  **(95% CI)** | **p value** |
| **Age** | 1.04 (1.02-1.06) | <0.001 | 1.04 (1.02-1.07) | <0.001 | 1.04 (1.02-1.06) | <0.001 | 1.02 (1.00-1.05) | 0.007 |
| **Sex, female** | 0.46 (0.34-0.64) | <0.001 |  |  | 0.43 (0.30-0.62) | <0.001 | 0.45 (0.31-0.66) | <0.001 |
| **Body mass index** |  |  |  |  |  |  |  |  |
| **Hypertension** |  |  |  |  |  |  |  |  |
| **Diabetes** |  |  |  |  |  |  |  |  |
| **Dyslipidemia** |  |  |  |  |  |  |  |  |
| **TDF (vs. ETV)** | 0.70 (0.46-1.07) | 0.10 |  |  |  |  |  |  |
| **HBeAg positivity** |  |  |  |  |  |  |  |  |
| **Elevated ALT** |  |  |  |  |  |  |  |  |
| **FIB-4** | 1.06 (1.03-1.10) | <0.001 |  |  | 1.06 (1.03-1.10) | <0.001 |  |  |
| **Undetectable HBV DNA levels**  **at one year** | 0.67 (0.51-0.89) | 0.006 |  |  |  |  |  |  |
| **HBeAg loss/seroconversion**  **at one year** |  |  | 0.88 (0.53-1.46) | 0.62 |  |  |  |  |
| **Normalization of ALT levels**  **at one year** |  |  |  |  | 0.84 (0.62-1.12) | 0.24 |  |  |
| **Improvement of FIB-4 index**  **at one year** |  |  |  |  |  |  | 0.60 (0.41-0.88) | 0.01 |

**Supplementary Table S6.** Factor associated with hepatocellular carcinoma among patients with elevated serum alanine aminotransferase levels (n = 747).

|  | **Un-adjusted HR (95% CI)** | **p value** | **Adjusted HR**  **(95% CI)** | **p value** |
| --- | --- | --- | --- | --- |
| **Age** | 1.03 (1.02-1.05) | <0.001 | 1.04 (1.02-1.06) | <0.001 |
| **Sex, female** | 0.56 (0.40-0.79) | <0.001 | 0.43 (0.30-0.62) | <0.001 |
| **Body mass index** | 0.99 (0.92-1.06) | 0.79 |  |  |
| **Hypertension** | 1.01 (0.64-1.60) | 0.94 |  |  |
| **Diabetes** | 1.18 (0.76-1.83) | 0.44 |  |  |
| **Dyslipidemia** | 0.84 (0.46-1.55) | 0.59 |  |  |
| **TDF (vs. ETV)** | 0.62 (0.38-1.00) | 0.05 |  |  |
| **HBeAg positivity** | 1.31 (0.98-1.76) | 0.07 |  |  |
| **HBV DNA (log_10_ IU/ml)** | 1.07 (0.95-1.20) | 0.25 |  |  |
| **FIB-4 index** | 1.07 (1.03-1.10) | <0.001 | 1.06 (1.03-1.10) | <0.001 |
| **Normalization of ALT levels at one year** | 0.88 (0.65-1.17) | 0.39 | 0.82 (0.61-1.10) | 0.19 |

**Supplementary Table S7.** Factor associated with hepatocellular carcinoma among patients with elevated FIB-4 index (n = 478).

|  | **Un-adjusted HR (95% CI)** | **p value** | **Adjusted HR**  **(95% CI)** | **p value** |
| --- | --- | --- | --- | --- |
| **Age** | 1.02 (1.00-1.04) | 0.031 | 1.03 (1.00-1.05) | 0.006 |
| **Sex, female** | 0.56 (0.39-0.80) | 0.002 | 0.45 (0.31-0.65) | <0.001 |
| **Body mass index** | 0.99 (0.93-1.06) | 0.85 |  |  |
| **Hypertension** | 0.91 (0.53-1.56) | 0.74 |  |  |
| **Diabetes** | 1.15 (0.73-1.79) | 0.53 |  |  |
| **Dyslipidemia** | 0.99 (0.48-2.04) | 0.99 |  |  |
| **TDF (vs. ETV)** | 0.60 (0.33-1.06) | 0.08 |  |  |
| **HBeAg positivity** | 1.00 (0.71-1.39) | 0.99 |  |  |
| **HBV DNA (log_10_ IU/ml)** | 0.98 (0.86-1.12) | 0.81 |  |  |
| **Elevated ALT level** | 0.86 (0.56-1.32) | 0.50 |  |  |
| **Improvement of FIB-4 index (<3.25) at one year** | 0.59 (0.40-0.86) | 0.007 | 0.55 (0.38-0.82) | 0.003 |

|  | **Number at risk** | **5-year HCC incidence rates** | **P value** |
| --- | --- | --- | --- |
| **Modified PAGE-B score <=8** | 51 |  | 0.61 |
| Without improvement of FIB-4 | 1 | 0% |  |
| With improvement of FIB-4 | 50 | 6.2% |  |
| **Modified PAGE-B score <=8** | 305 |  | 0.005 |
| Without improvement of FIB-4 | 91 | 18.0% |  |
| With improvement of FIB-4 | 305 | 9.4% |  |
| **Modified PAGE-B score <=8** | 464 |  | 0.005 |
| Without improvement of FIB-4 | 259 | 29.0% |  |
| With improvement of FIB-4 | 205 | 19.3% |  |

**Supplementary Table S8.** 5-year HCC incidence rates according to FIB-4 at one year after antiviral treatment in modified PAGE-B subgroups
